# Supplementary material for: Modeling glioblastoma heterogeneity as a dynamic network of cell states
Source: Mol Syst Biol. 2021 Sep 16;17(9):e10105. doi: 10.15252/msb.202010105 (PMC8444284; doi:10.15252/msb.202010105)
Supplement: Supplementary file 5 — Source Data for Figure 3 [file MSB-17-e10105-s001.zip › Figure3A_sourcedata/GSEA_3065/hallmarks_state4.GseaPreranked.1623416296068/HALLMARK_CHOLESTEROL_HOMEOSTASIS.html]

Details for gene set HALLMARK\_CHOLESTEROL\_HOMEOSTASIS[GSEA]

|  || Dataset | state4 |
| Phenotype | NoPhenotypeAvailable |
| Upregulated in class | na\_neg |
| GeneSet | HALLMARK\_CHOLESTEROL\_HOMEOSTASIS |
| Enrichment Score (ES) | -0.47527644 |
| Normalized Enrichment Score (NES) | -1.5160649 |
| Nominal p-value | 0.03709199 |
| FDR q-value | 0.050272297 |
| FWER p-Value | 0.485 |
Table: GSEA Results Summary

  

Fig 1: Enrichment plot: HALLMARK\_CHOLESTEROL\_HOMEOSTASIS      
 Profile of the Running ES Score & Positions of GeneSet Members on the Rank Ordered List

  

| PROBE | GENE SYMBOL | GENE\_TITLE | RANK IN GENE LIST | RANK METRIC SCORE | RUNNING ES | CORE ENRICHMENT || 1 | FABP5 |  |  | 23 | 0.375 | 0.0573 | No |
| 2 | PNRC1 |  |  | 30 | 0.354 | 0.1140 | No |
| 3 | LGALS3 |  |  | 89 | 0.259 | 0.1468 | No |
| 4 | CLU |  |  | 95 | 0.252 | 0.1869 | No |
| 5 | ECH1 |  |  | 245 | 0.187 | 0.1933 | No |
| 6 | TP53INP1 |  |  | 668 | 0.118 | 0.1447 | No |
| 7 | ANXA5 |  |  | 814 | 0.103 | 0.1381 | No |
| 8 | CPEB2 |  |  | 1626 | 0.058 | 0.0171 | No |
| 9 | TM7SF2 |  |  | 1689 | 0.056 | 0.0162 | No |
| 10 | ETHE1 |  |  | 1809 | 0.052 | 0.0055 | No |
| 11 | ACTG1 |  |  | 1933 | 0.049 | -0.0063 | No |
| 12 | HMGCS1 |  |  | 2674 | 0.031 | -0.1202 | No |
| 13 | IDI1 |  |  | 2749 | 0.030 | -0.1273 | No |
| 14 | ATXN2 |  |  | 3075 | 0.024 | -0.1757 | No |
| 15 | LPL |  |  | 3121 | 0.023 | -0.1792 | No |
| 16 | ABCA2 |  |  | 3231 | 0.020 | -0.1935 | No |
| 17 | CTNNB1 |  |  | 3329 | 0.017 | -0.2062 | No |
| 18 | PMVK |  |  | 3360 | 0.017 | -0.2084 | No |
| 19 | LSS |  |  | 3382 | 0.016 | -0.2091 | No |
| 20 | S100A11 |  |  | 3436 | 0.014 | -0.2154 | No |
| 21 | MVK |  |  | 3880 | -0.003 | -0.2861 | No |
| 22 | SEMA3B |  |  | 3975 | -0.008 | -0.2999 | No |
| 23 | FDFT1 |  |  | 4088 | -0.014 | -0.3156 | No |
| 24 | FASN |  |  | 4185 | -0.021 | -0.3276 | No |
| 25 | ATF3 |  |  | 4262 | -0.026 | -0.3356 | No |
| 26 | JAG1 |  |  | 4496 | -0.044 | -0.3659 | No |
| 27 | ALCAM |  |  | 4499 | -0.045 | -0.3589 | No |
| 28 | SCD |  |  | 4717 | -0.061 | -0.3839 | No |
| 29 | SREBF2 |  |  | 5022 | -0.084 | -0.4191 | No |
| 30 | PLAUR |  |  | 5171 | -0.099 | -0.4268 | No |
| 31 | CYP51A1 |  |  | 5190 | -0.101 | -0.4133 | No |
| 32 | GPX8 |  |  | 5326 | -0.111 | -0.4169 | No |
| 33 | FADS2 |  |  | 5544 | -0.135 | -0.4298 | No |
| 34 | FDPS |  |  | 5828 | -0.190 | -0.4444 | Yes |
| 35 | LDLR |  |  | 5843 | -0.194 | -0.4152 | Yes |
| 36 | HMGCR |  |  | 5906 | -0.208 | -0.3912 | Yes |
| 37 | ACAT2 |  |  | 5931 | -0.215 | -0.3601 | Yes |
| 38 | GUSB |  |  | 6024 | -0.247 | -0.3347 | Yes |
| 39 | DHCR7 |  |  | 6083 | -0.287 | -0.2973 | Yes |
| 40 | SQLE |  |  | 6123 | -0.316 | -0.2521 | Yes |
| 41 | TMEM97 |  |  | 6131 | -0.324 | -0.2006 | Yes |
| 42 | EBP |  |  | 6133 | -0.324 | -0.1481 | Yes |
| 43 | CD9 |  |  | 6176 | -0.378 | -0.0933 | Yes |
| 44 | TNFRSF12A |  |  | 6251 | -0.658 | 0.0019 | Yes |
Table: GSEA details [plain text format]

  

Fig 2: HALLMARK\_CHOLESTEROL\_HOMEOSTASIS: Random ES distribution      
 Gene set null distribution of ES for **HALLMARK\_CHOLESTEROL\_HOMEOSTASIS**
